# Efficacy and safety of Lianhua Qingwen as an adjuvant treatment for influenza in Chinese patients: A meta-analysis

**DOI:** 10.1097/MD.0000000000036986

**Published:** 2024-01-19

**Authors:** Chao Yuan, Ying Guan

**Affiliations:** a Department of Pharmacy, Weifang People’s Hospital, Weifang, China; b Department of Medical Insurance Office, Weifang People’s Hospital, Weifang, China.

**Keywords:** clinical effective rate, cure rate, FLu, LHQW, randomized controlled trials

## Abstract

**Background:**

Lianhua Qingwen (LHQW) is a proprietary traditional Chinese medicine for the treatment of influenza (FLu). It is composed of 2 prescriptions, Maxing Shigan and Yinqiao, which has antiviral, antibacterial, and immunomodulatory effects. However its clinical suitability has not yet been investigated.

**Objective:**

This study aimed to evaluate the efficacy and safety of LHQW in the treatment of FLu.

**Methods:**

We searched several databases, including PubMed and China Biomedical Database for literature research, from inception to July 1, 2023. This meta-analysis included RCTs that compared the safety and efficacy of the combination of LHQW and conventional drugs (CD) with CD alone for IFU. The extracted data were analyzed using Revman5.4 software with risk ratio (RR), 95% confidence intervals (CI), and standardized mean difference.

**Results:**

Our meta-analysis included 32 articles with 3592 patients. The results showed that the effects of LHQW adjuvant therapy were superior to those of CD (clinical effective rate: RR = 1.22, 95% CI: 1.18–1.26, *P* < .00001; cure rate: RR = 1.54, 95% CI: 1.35–1.75, *P* < .00001), and adverse reactions after treatment were significantly lower than those before treatment (RR = 0.70, 95% CI: 0.50–0.98, *P* = .04).

**Conclusion:**

This meta-analysis indicates that LHQW combined with CD may be more effective than CD alone for the treatment of FLu.

## 1. Introduction

Influenza (FLu) is an acute respiratory infectious disease caused by the influenza virus. It is mainly transmitted through droplets in the air and indirectly through virus-contaminated objects via the general susceptibility of the population and as a seasonal epidemic characterized by high infectivity, rapid transmission, and high incidence.^[1–3]^ Influenza viruses are classified into 4 types, namely A, B, C, and D, according to the antigenicity of viral matrix protein 1 (matrix1, M1) and nucleoprotein. The influenza A virus can be divided into different subtypes according to the molecular characteristics of its surface glycoprotein hemagglutinin and neuraminidase.^[4,5]^ To date, 18 hemagglutinin isoforms (H1–H18) and 11 neuraminidase isoforms (N1–N11) have been identified that can result in serious respiratory diseases in humans and pandemics worldwide. It is estimated that the disease can cause 290,000 to 650,000 deaths worldwide each year due to respiratory-related diseases alone.^[6]^ Although influenza is mostly self-limiting, many patients develop critical illnesses, such as pneumonia, respiratory failure, multi-organ failure, and even death,^[7]^ resulting in personal health risks and public health burden.^[8]^

For thousands of years, traditional Chinese medicine has been widely used for the prevention and treatment of diseases.^[9,10]^ In 2003, Lianhua Qingwen (LHQW) was commonly used to treat severe acute respiratory syndrome.^[11]^ In recent decades, it has been widely used for the treatment of viral influenza, pneumonia caused by coronavirus, the common cold, and other diseases.^[12,13]^ LHQW is derived from 2 formulas: Ma Xing Shi Gan and Yin Qiao San.^[14,15]^ The following 13 herbal ingredients are present in LHQW: *Isatis indigotica* Fortune ex Lindl., *Forsythia suspensa* (Thunb.) Vahl., *Lonicera japonica* Thunb., *Dryopteris crassirhizoma* Nakai, *Ephedra sinica* Stapf, *Armeniaca Sibirica* (L.) Lam., *Houttuynia cordata* Thunb, *Pogostemon cablin* (Blanco) Benth., *Rhodiola rosea* L., *Rheum officinale* Baill, *Glycyrrhiza uralensis* Fisch., *Mentha haplocalyx* Briq, and Plaster.^[12,16]^ The main active ingredients of LHQW have also been identified: quercetin, kaempferol, lignan, β-sitosterol, indigo, baicalein, tryptam, [E]-4-phenyl-3-buten-2-tone, 1-methyl-2-nonyl-4(1H)-quinolone, stigmasterol, naringenin, and 18β-glycyrrhetinic acid.^[16]^ These major active components act on multiple targets and pathways,^[17,18]^ and are involved in T cell activation, viral receptors, and inflammatory response pathways associated with antiviral and anti-inflammatory responses.^[19]^

The basic principle of Chinese medicine in influenza is to remove superficial evils, promote lung, relieve superficial symptoms and take care of concurrent symptoms. Because there are differences in the nature of dampness, summer, heat, and cold, the method of relieving the epidermis, such as dissolving dampness, clearing summer, pungent coolness, and pungent warmth, is often used in clinical treatment according to the patient’s condition. In Chinese medicine, influenza belongs to the category of exogenous warm-heat disease, and LHQW treats both the exterior and interior, clears distemper, detoxifies, promotes the lung, and releases heat, and has various effects such as antiviral, anti-inflammatory, antipyretic, immune regulation.^[20]^ Some studies have shown that it has a good therapeutic effect on influenza; however, the sample size included in each study was limited, and such experimental results were not objective and comprehensive enough, thereby affecting the overall credibility of the efficacy of the combination; furthermore, the scope of clinical promotion and application was not ideal. In this study, we conducted a meta-analysis by collecting clinical randomized controlled trials (RCTs) of adjuvant treatment of influenza with LHQW, aiming to provide a scientific reference to promote the design and implementation of influenza and provide a theoretical basis for its clinical application.

## 2. Materials and methods

This systematic review and meta-analysis were performed based on the Preferred Reporting Items for Systematic Reviews and Meta-Analyses guidelines. Our meta-analysis was registered in the International prospective register of systematic reviews (PROSPERO registration number: CRD42023388407).

### 2.1. Search strategy

A literature search was conducted in PubMed, EMBASE, Web of Science, Cochrane Library, China Knowledge Infrastructure (CNKI), Wanfang, Weipu, and China Biomedical Database (CBM). RCTs were obtained using the following keywords: Lianhua Qingwen, Lianhua Qingwen capsule, Lianhua Qingwen granules, influenza, FLu, H1N1, H3N2, and H7N9. The search period was from the establishment of the database to July 1, 2023.

### 2.2. Eligibility criteria

#### 2.2.1. Inclusion criteria.

1. Patients who met the diagnostic criteria for confirmed or suspected influenza, including symptoms and signs, past history, and laboratory tests, regardless of disease severity, age, sex and race of the participants.
2. Patients receiving conventional drugs (CD) (control group) or LHQW in combination with CD (experimental group).
3. Reported efficacy or safety data of LHQW against influenza: primary outcomes included clinical effectiveness, and cure rate; secondary outcomes included time to fever resolution, time to cough disappearance, C-reactive protein (CRP), CD4+/CD8+, and adverse effects.
4. Only RCTs were included.

#### 2.2.2. Exclusion criteria.

1. No RCTs or influenza combined with other diseases.
2. Inappropriate standards of experimental group or control group; in addition to LHQW, the experimental group also contained other treatment measures that the control group did not.
3. Incomplete or invalid data.
4. Non-clinical studies, review papers, meta-analyses, conference abstracts, case reports, and graduation papers.

### 2.3. Outcome definitions

The primary outcome indicators included the clinical effective rate and cure rate, while the secondary outcome indicators included time to disappearance of fever, cough, sore throat, muscle aches, runny nose, nasal congestion, headache, time to virus conversion, hospitalization time, time to improvement of systemic symptoms, time to improvement of respiratory symptoms, CRP, interleukin (IL)-6, tumor necrosis factor (TNF)-α, CD4^+^/CD8^+^, and adverse drug reactions (ADRs). Cured: the main clinical symptoms completely disappeared and the body temperature returned to normal; Ineffectiveness: the disease-related symptoms and signs did not change significantly or even worsened. Clinical efficacy rate = (total number − ineffective number)/total number. Cure rate = number of cured patients/total number of patients.

### 2.4. Data extraction and quality assessment

The following data were extracted from the eligible literature: first author’s name, year of publication, number of cases, age of patients, intervention method, dose of LHQW, duration of treatment, observed indicators, and ADR.

The final articles included in this analysis were independently screened by 2 reviewers. After excluding duplicate studies, the titles and abstracts of the remaining articles were reviewed. The full text of the remaining studies was independently reviewed by 2 other reviewers. When the 2 reviewers disagreed on the final article, a third reviewer resolved the dispute.

According to the risk of bias assessment recommended by RevMan 5.4, the assessment criteria included 7 dimensions of evaluation items: (a) random sequence generation (selection bias), (b) allocation concealment (selection bias), (c) blinding of participants and personnel (performance bias), (d) blinding of outcome assessment (detection bias), (e) incomplete outcome data (attrition bias), (f) selective reporting (reporting bias), and (g) other biases.

The Cochrane Correspondence Network RCT assessment tool was used to assess each project based on low risk (−), unknown risk (?), and high risk (+). The quality of the literature was evaluated through group discussion.

### 2.5. Statistical methods

RevMan5.4 software was used for statistical analysis of the data, and the relative risk (RR) and 95% confidence interval (CI) of the dichotomous variables were used as effect indicators for this study, while standardized mean difference (SMD) and CI were used as continuous variables. *I*^2^ and *P* values were used as indicators for the heterogeneity test. If there was no significant heterogeneity in the combined data (*P* > .10, *I*^2^ < 50%), a fixed-effects model was used; when there was significant heterogeneity (*P* < .10, *I*^2^ > 50%), a random-effects model was used. The difference was considered statistically significant when the *P* value was <.05. Funnel plots were used to analyze the presence of publication bias.

### 2.6. Ethical review

The protocol does not require ethics committee review because this study is based on published literatures.

## 3. Results

### 3.1. Search results

**Figure 1.**
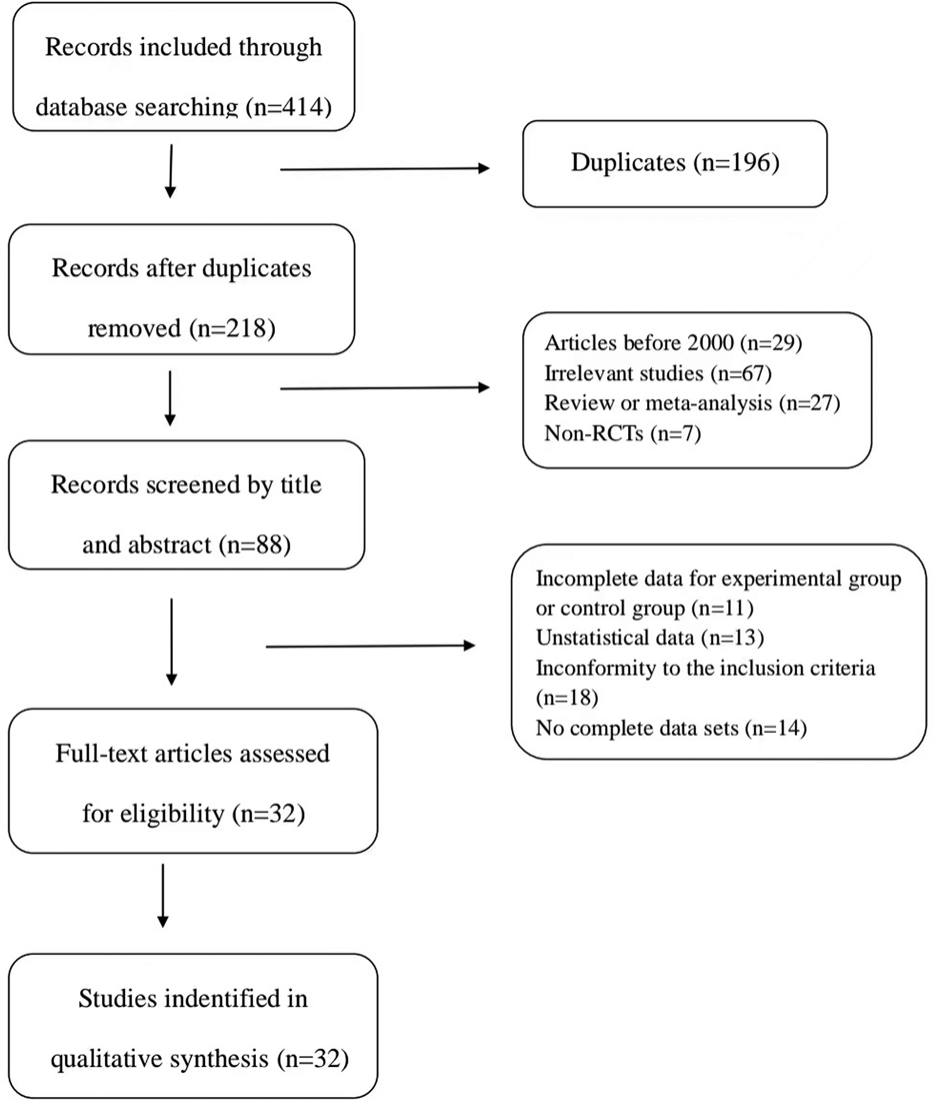
Literature screening process and results.

Based on the search strategy, 414 relevant studies were included in the analysis. Among them, 7 articles were included in PubMed, 3 articles in EMBASE, 4 articles in Web of Science, 1 article in Cochrane Library, 73 articles in CBM, 143 articles in CNKI, 117 articles in WanFang, and 66 articles in VIP. After removing duplicate articles and studies, 218 articles were retained. Subsequently, 130 articles were excluded by reading the titles and abstracts of the articles, and after further reading the full text, 32 eligible articles^[21–52]^ were finally included in our meta-analysis (Fig. 1).

### 3.2. Basic characteristics

A total of 3592 subjects were included in the 32 studies, all of whom were influenza patients, comprising 1802 individuals in the experimental group and 1790 in the control group. The experimental group received LHQW combined with CD, whereas the control group received only CD. Randomization was performed in all the 32 studies, with a maximum sample size of 150 and a minimum of 15 in a single randomized controlled trial. LHQW granules (6 g/sachet, Yiling Pharmaceutical, Shijiazhuang, China) were used in 16 studies,^[23,24,27,28,30,31,35–37,39,42–46,52]^ LHQW capsules (0.35 g/capsule, Yiling Pharmaceutical, Shijiazhuang, China) were used in 10 studies,^[21,25,26,32–34,38,41,47,50]^ while 6 studies^[22,29,40,48,49,51]^ did not present manufacturer information regarding LHQW (Table 1).

**Table 1:** Characteristics of the included studies.

Included studies	Simple size (Exp/Con)	Age	Interventions measure (Exp/Con)	LHQW dosage	Time	Outcomes
Chen LY 2017	47/47	18–65	LHG + OVC/OVC	4 capsules, qd 75 mg, bid	5d	,-
Chen N 2022	50/50	18–23	LHC + OVC/OVC	1.4 g, qd 75 mg, bid	7d	
Cui Y 2021	77/70	19–60	LHG + OVC/OVC	6 g, tid 75 mg, bid	5d	-,
Du FL 2019	30/30	NG	LHG + OVG/OVG	6 g, tid 75 mg, bid	5d	
Hao Y 2021	60/60	20–60	LHC + OVC/OVC	4 capsules, tid 75mg, bid	7d	
Hou X 2021	60/60	20–50	LHC + OVC/OVC	4 capsules, tid 75mg, bid	3d	-,
Hua L 2019	51/51	18–60	LHG + OVC/OVC	6g, tid 1 capsules, bid	5d	-,
Huang JH 2017	100/100	18–48	LHC + RibI/RibI	4 capsules, tid 10 mg/kg, bid	3d	
Huang ZQ 2020	75/75	3–12	LHG + OVC/OVC	1/3–1 granule, tid 75 mg, bid	5d	-,
Lei X 2020	49/48	4–13	LHG + OVG/OVG	3–6 g, bid 30–75 mg, bid	5d	-,
Liang ZS 2019	49/49	1–14	LHC + OVC/OVC	4 capsules, tid 60mg, bid	7d	-,
Li G 2016	63/63	24–34	LHC + OVC/OVC	4 capsules, tid 45–75 mg, bid	3–7d	-,
Li SX 2018	43/40	1–12	LHG + OVC/OVC	6g, tid 30–75 mg, bid	5d	-,
Liu Y 2020	34/34	3–6	LHG + OVC/OVC	3–6 g, tid 30–60 mg, bid	7d	,-,,-
Liu ZN 2020	30/30	1–8	LHG + OVG/OVG	3–6 g, tid 30–60 mg, bid	5d	
Li ZW 2021	140/140	24–29	LHC + OVC/OVC	4 capsules, tid 75 mg, tid	3d	
Peng YF 2019	150/150	12–80	LHG + OVC/OVC	1.05 g, tid 45–75 mg, bid	7d	-,
Qian XK 2019	47/47	18–80	LHC + OVC/OVC	1.05 g, tid 45–75 mg, bid	7d	
Shen NN 2021	36/35	1–8	LHG + REG/REG	1–2 g, tid 10 mg/kg, qid	7d	
Sun SG 2014	15/15	25–29	LHC + OVC/OVC	2–4 capsules, tid 45–75mg, bid	5–7d	
Wang Y 2020	62/62	3–12	LHC + OVG/OVG	4 capsules, tid NG	3d	
Wei F 2022	30/30	19–78	LHG + OVC/OVC	6 g, tid 75 mg, bid	5d	-,-,
Xu ML 2021	46/46	18–65	LHG + OVG/OVG	6 g, tid 75 mg, bid	5d	
Ye GX 2021	75/75	3–13	LHG + OVG/OVG	3–6 g, tid 30–60 mg, bid	7d	
Yi W 2020	30/30	16–67	LHG + OVC/OVC	6 g, tid 75mg, bid	5d	,-
Yu CM 2018	41/41	3–14	LHG + OVG/OVG	6g, tid 30–75mg, bid	5–7d	
Zhang J 2019	40/40	22–67	LHC + OVC/OVC	4 capsules, tid 45–75 mg, bid	5–7	
Zhang JH 2021	30/30	5–64	LHC + OVC/OVC	4 capsules, tid 15–75 mg, bid	NG	
Zhang LH 2021	62/62	18–60	LHG + OVC/OVC	6 g, tid 75 mg, bid	5d	-,
Zhang RY 2020	45/45	23–80	LHC + OVC/OVC	1.4 g, qd 75 mg, bid	7d	
Zhou JL 2018	25/25	26–31	LHC + OVC/OVC	2–4 capsules, tid 45–75 mg, bid	5–7d	
Zhu SJ 2019	110/110	3–13	LHG + OVC/OVC	3–6 g, tid 30–75 mg, tid	3d	

Con = control group, Exp = experimental group, LHG = Lianhuaqingwen Granules, LHC = Lianhuaqingwen Capsules, LHQW = Lianhua Qingwen , OVC = Oseltamivir capsules, OVG = Oseltamivir Granules, REG = Ribavirin effervescent granules, RibI = Ribavirin injection.

Clinical efficacy rate; Cure rate; Time to relief from fever; Time to relief from cough; Time to relief from sore throat; Time to relief from muscle pain; Time to relief from runny nose; Time to relief from nasal congestion; Time to relief from headache; Time to virus extinction; Time to improvement in systemic symptom; Time to improvement in respiratory symptom; Length of hospital stay; CRP improvement; IL-6 improvement; TNF-α improvement; CD4^+^/CD8^+^ improvement; Adverse drugs reaction.

### 3.3. Quality evaluation

**Figure 2.**
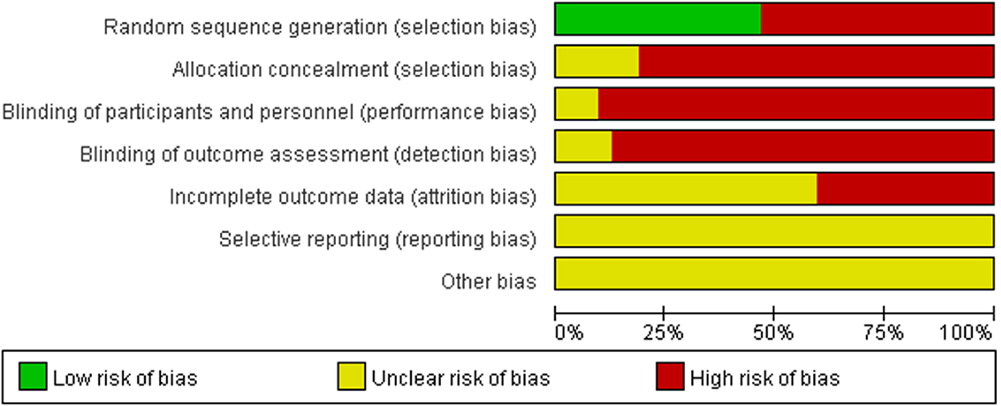
Risk of bias graph.

**Figure 3.**
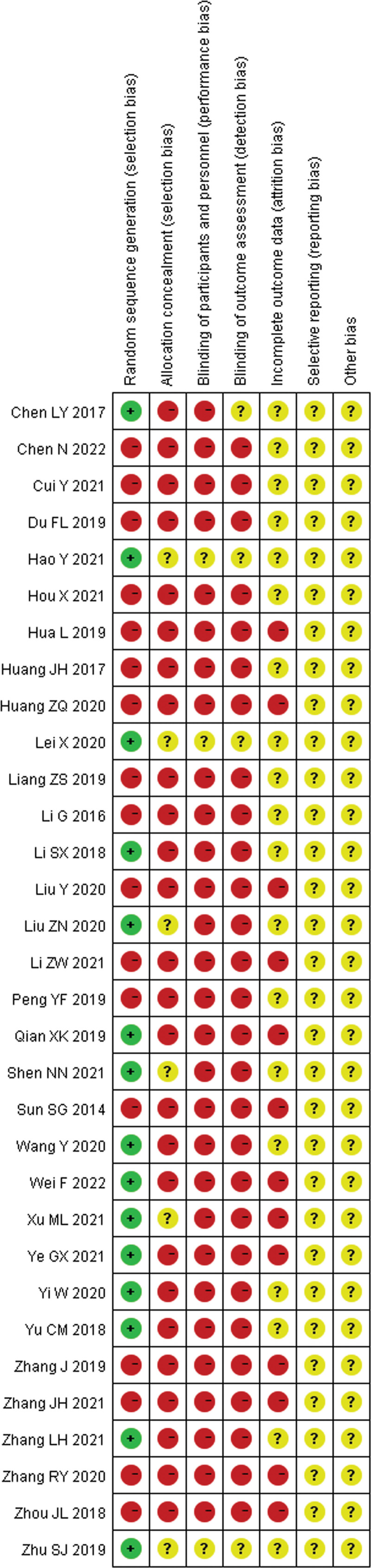
Risk of bias summary.

Among the 32 included studies, 15^[22,25,30,31,36,38,39,41–46,49,52]^ that described a randomized approach were determined as low-risk, and the other 17 studies did not specifically describe the randomization process. None of the studies in this analysis provided a clear description of allocation concealment, implementation bias, measurement bias, reporting bias, or other biases. In addition, 13 studies^[27,28,32,35,38,40,42–44,47–49,51]^ reported the identified bias as high-risk because of the lack of specific outcome data (Figs. 2 and 3).

### 3.4. Outcome measures

#### 3.4.1. Clinical efficacy rate.

**Figure 4.**
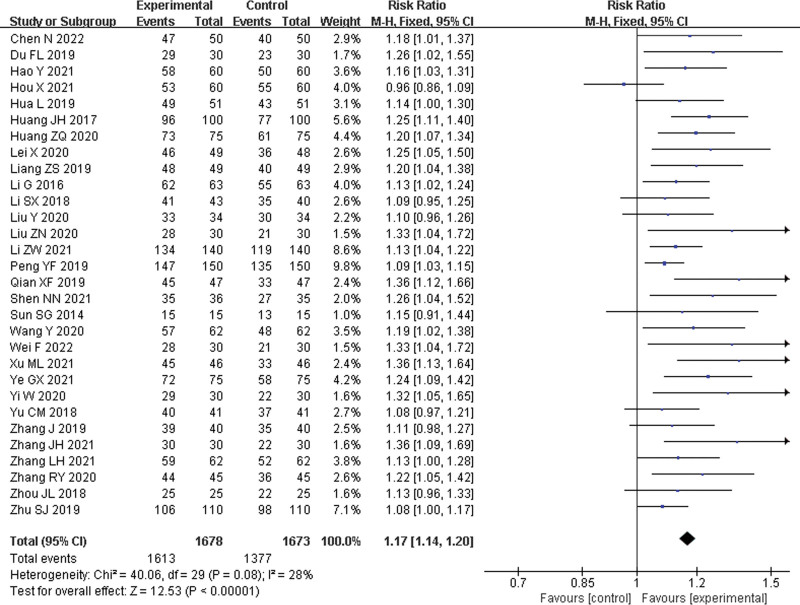
Clinical efficacy rate.

Thirty studies^[21,24–52]^ were included, with 1678 patients in the experimental group and 1673 in the control group. The statistical heterogeneity between the results of the studies was low (*P* = .08, *I*^2^ = 28%), and a random-effects model was performed. The results showed a significant difference between the 2 groups, and the experimental group with LHQW improved the clinical efficacy rate compared to the control group (RR = 1.16, 95% CI: 1.12–1.19, *P* < .00001) (Fig. 4).

#### 3.4.2. Cure rate.

**Figure 5.**
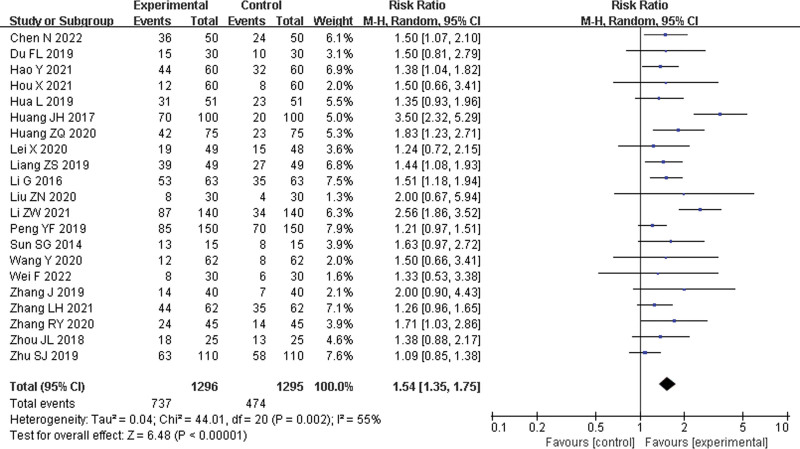
Cure rate.

Twenty-one studies^[21,24–30,32–34,36,37,40–42,47–49,51,52]^ were included, with 1296 patients in the experimental group and 1295 in the control group. There was high statistical heterogeneity between the results of the studies (*P* = .002, *I*^2^ = 55%), and a random effects model was used for the analysis. The results showed a significant difference between the experimental group and control group, and the experimental group showed a better cure rate (RR = 1.54, 95% CI: 1.35–1.75, *P* < .00001) (Fig. 5).

#### 3.4.3. Time to relief from fever.

**Figure 6.**
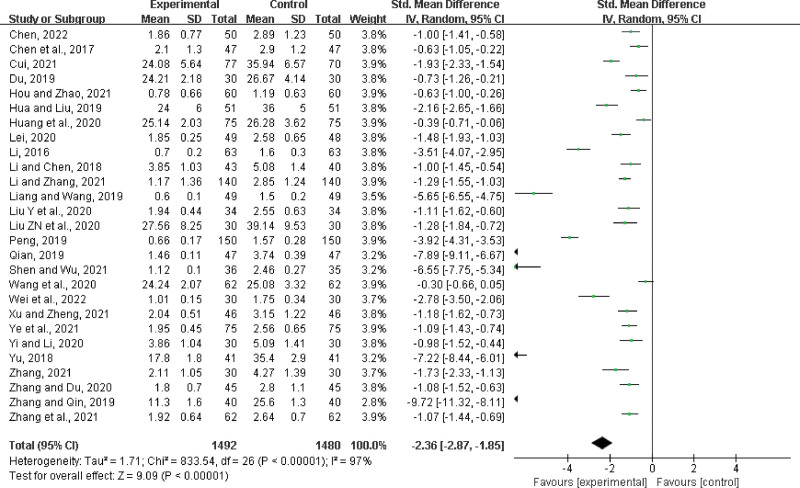
Time to relief from fever.

Twenty-seven^[21–24,26–28,30–39,41–50]^ studies were included, with 1492 patients in the experimental group and 1480 in the control group. There was a statistically significant heterogeneity between the results of the studies (*P* < .00001, *I*^2^ = 97%), and a random-effects model was applied. The results showed a significant difference between the 2 groups, and the experimental group with LHQW had a shortened time to fever relief caused by influenza (SMD = −2.36, 95% CI: −2.87 to −1.85, *P* < .00001) (Fig. 6).

#### 3.4.4. Time to relief from cough.

**Figure 7.**
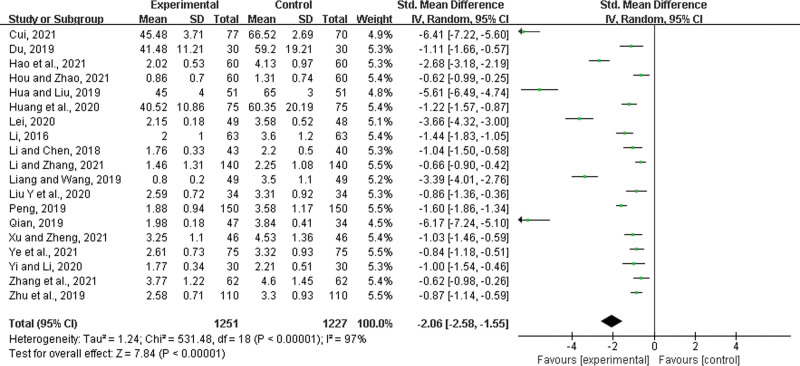
Time to relief from cough.

Nineteen studies^[23–28,30–35,37,38,43–45,49,52]^ were included, with 1251 patients in the experimental group and 1227 in the control group. There was a statistically significant heterogeneity between the results of the studies (*P* < .00001, *I*^2^ = 97%), and a random-effects model was performed. The results showed a significant difference between the 2 groups, and that the experimental group with LHQW could shorten the time to cough relief (SMD = 2.06, 95% CI: −2.58 to −1.55, *P* < .00001) (Fig. 7).

#### 3.4.5. Time to relief from sore throat.

**Figure 8.**
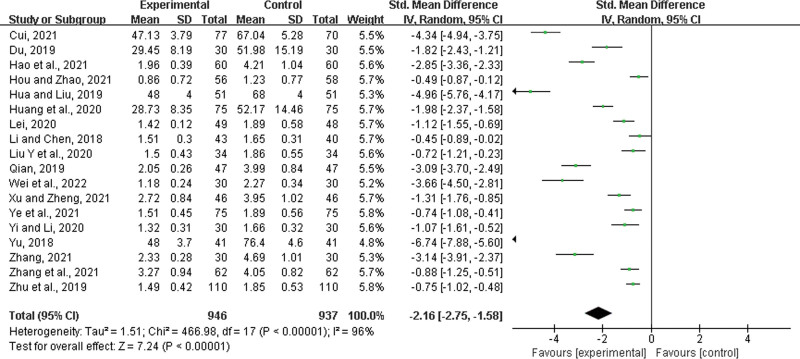
Time to relief from sore throat.

Eighteen studies^[23–28,30,31,35,38,42–46,49,50,52]^ were included, with 946 participants in the experimental group and 937 in the control group. There was statistically significant heterogeneity among the results (*P* < .00001, *I*^2^ = 96%), and a random effects model was used for the meta-analysis. The results showed a significant difference between the 2 groups. In the experimental group, LHQW shortened the time to relief from sore throat (SMD = −2.16, 95% CI: −2.75 to −1.58, *P* < .00001) (Fig. 8).

#### 3.4.6. Time to relief from muscle pain.

**Figure 9.**
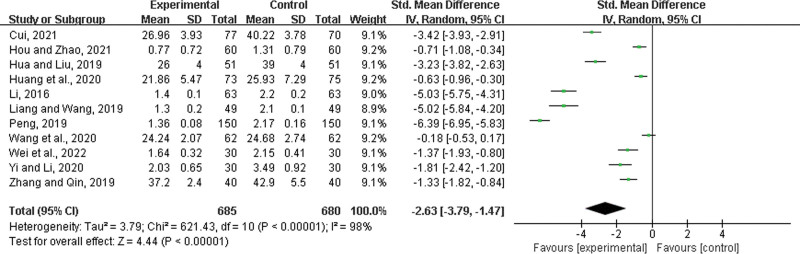
Time to relief from muscle pain.

Eleven studies^[23,26–28,33,34,37,41,42,45,48]^ were included, with 685 patients in the experimental group and 680 in the control group. There was a statistically significant heterogeneity among the results (*P* < .00001, *I*^2^ = 98%), and a random effects model was used for the meta-analysis. The results showed a significant difference between the 2 groups. In the experimental group, LHQW shortened the time to relief from muscle pain (SMD = −2.63, 95% CI: −3.79 to −1.47, *P* < .00001) (Fig. 9).

#### 3.4.7. Time to relief from runny nose.

**Figure 10.**
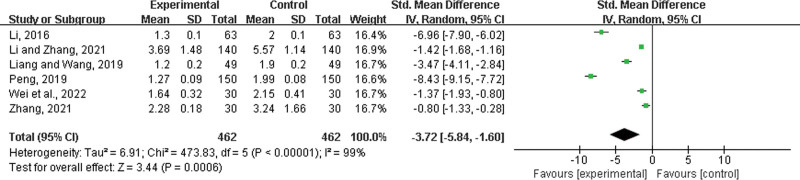
Time to relief from runny nose.

Six studies^[32–34,37,42,50]^ were included, both with 462 patients in the experimental group and control group. There was a statistically significant heterogeneity among the results (*P* < .00001, *I*^2^ = 99%), and a random effects model was used for the meta-analysis. The results showed a significant difference between the 2 groups, and in the experimental group, LHQW shortened the time to relief from runny nose (SMD = −3.72, 95% CI: −5.84 to −1.60, *P* = .0006) (Fig. 10).

#### 3.4.8. Time to relief from nasal congestion.

**Figure 11.**
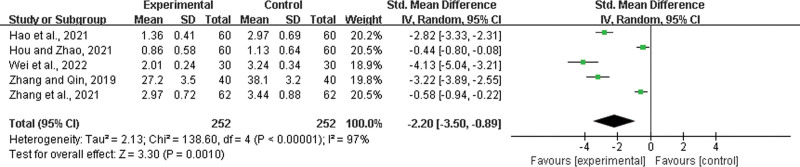
Time to relief from nasal congestion.

Five studies^[25,26,42,48,49]^ were included, both with 252 patients in the experimental group and control group. There was a statistically significant heterogeneity among the results (*P* < .00001, *I*^2^ = 97%), and a random effects model was used for the meta-analysis. The results showed a significant difference between the 2 groups, and the experimental group had a shortened time to relief from nasal congestion (SMD = −2.20, 95% CI: −3.50 to −0.89, *P* = .0010) (Fig. 11).

#### 3.4.9. Time to relief from headache.

**Figure 12.**
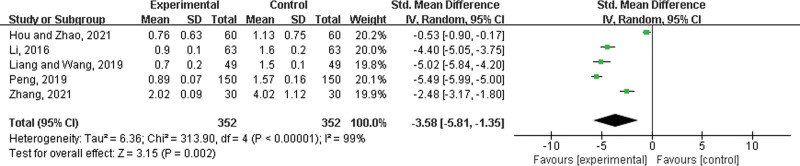
Time to relief from headache.

Five studies^[26,33,34,37,50]^ were included, both with 352 patients in the experimental group and control group. There was a statistically significant heterogeneity among the results (*P* < .00001, *I*^2^ = 99%), and a random effects model was used for the meta-analysis. The results showed a significant difference between the 2 groups. In the experimental group, LHQW shortened the time to relief from headache (SMD = −3.58, 95% CI: −5.81 to −1.35, *P* = .002) (Fig. 12).

#### 3.4.10. Time to virus extinction.

**Figure 13.**
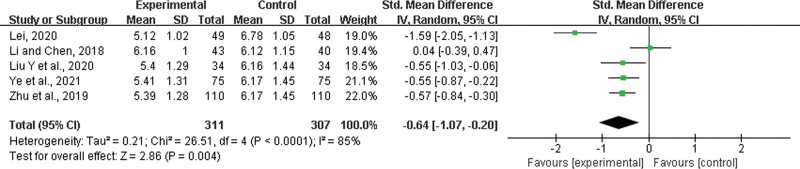
Time to virus extinction.

Five studies^[30,31,35,44,52]^ were included, with 311 patients in the experimental group and 307 in the control group. There was a statistically significant heterogeneity among the results of each study (*P* < .0001, *I*^2^ = 85%), and a random effects model was used for the meta-analysis. The results showed a significant difference between the 2 groups. The experimental group with LHQW could shorten the time to virus extinction (SMD = −0.64, 95% CI: −1.07 to −0.20, *P* = .004) (Fig. 13).

#### 3.4.11. Time to improvement in systemic symptom.

**Figure 14.**
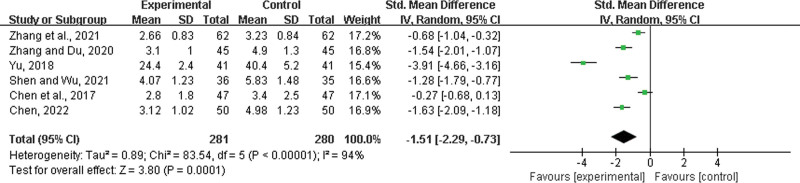
Time to improvement in systemic symptom.

Six studies^[21,22,39,46,47,49]^ were included, with 281 patients in the experimental group and 280 in the control group. There was a statistically significant heterogeneity among the results (*P* < .00001, *I*^2^ = 94%), and a random effects model was used for the meta-analysis. The results showed a significant difference between the 2 groups. The experimental group with LHQW had a shortened time to improvement in systemic symptom (SMD = −1.51, 95% CI: −2.29 to −0.73, *P* = .0001) (Fig. 14).

#### 3.4.12. Time to improvement in respiratory symptom.

**Figure 15.**
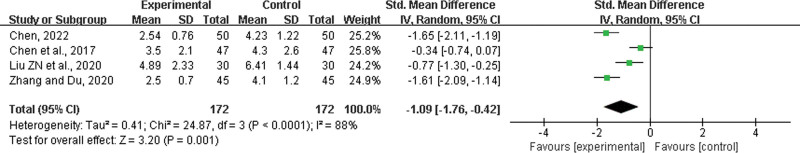
Time to improvement in respiratory symptom.

Four studies^[21,22,36,47]^ were included, both with 172 participants in the experimental group and control group. There was a statistically significant heterogeneity among the results (*P* < .00001, *I*^2^ = 88%), and a random effects model was used for the meta-analysis. The results showed a significant difference between the 2 groups, and that the experimental group had a shortened time to improvement in respiratory symptom (SMD = −1.09, 95% CI: −1.76 to −0.42, *P* = .001) (Fig. 15).

#### 3.4.13. Length of hospital stay.

**Figure 16.**
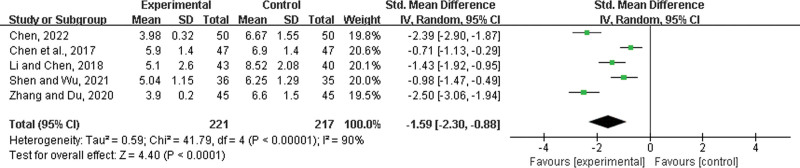
Length of hospital stay.

Five studies^[21,22,31,39,47]^ were included, with 221 patients in the experimental group and 217 in the control group. There was a statistically significant heterogeneity among the results (*P* < .00001, *I*^2^ = 90%), and a random effects model was used for the meta-analysis. The results showed a significant difference between the 2 groups. In the experimental group, LHQW shortened the length of hospital stay (SMD = −1.59, 95% CI: −2.30 to −0.88, *P* < .00001) (Fig. 16).

#### 3.4.14. CRP improvement.

**Figure 17.**
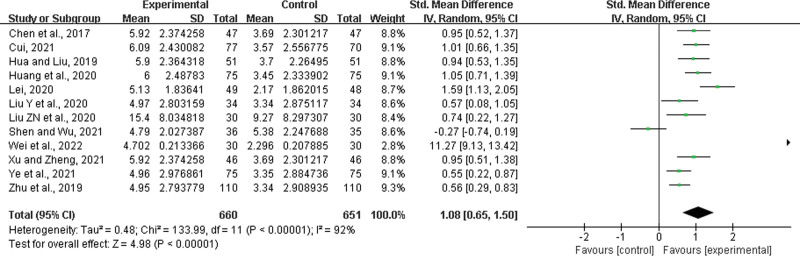
CRP improvement. CRP = C-reactive protein.

Twelve studies^[22,23,27,28,30,35,36,39,42–44,52]^ were included, with 660 patients in the experimental group and 651 in the control group. There was a statistically significant heterogeneity among the results (*P* < .00001, *I*^2^ = 92%), and a random effects model was used for the meta-analysis. The results showed a significant difference between the 2 groups, and in the experimental group, LHQW significantly improved CRP levels (SMD = 1.08, 95% CI: 0.65 to 1.50, *P* < .00001) (Fig. 17).

#### 3.4.15. IL-6 improvement.

**Figure 18.**
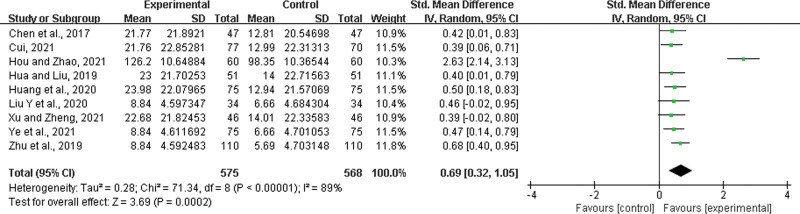
IL-6 improvement. IL = interleukin.

Nine studies^[22,23,26–28,35,43,44,52]^ were included, with 575 patients in the experimental group and 568 in the control group. There was a statistically significant heterogeneity among the results (*P* < .00001, *I*^2^ = 89%), and a random effects model was used for the meta-analysis. The results showed a significant difference between the 2 groups. In the experimental group, LHQW significantly improved IL-6 levels (SMD = 0.69, 95% CI: 0.32 to 1.05, *P* = .0002; Fig. 18).

#### 3.4.16. TNF-α improvement.

**Figure 19.**
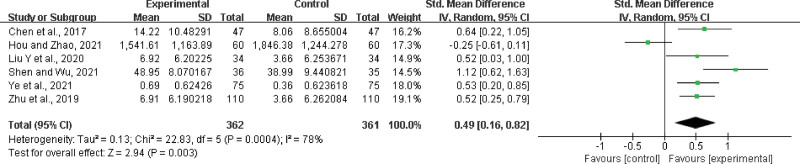
TNF-α improvement. TNF = tumor necrosis factor.

Six studies^[22,26,35,39,44,52]^ were included, with 362 participants in the experimental group and 361 in the control group. There was a statistically significant heterogeneity among the results (*P* = .0004, *I*^2^ = 78%), and a random effects model was used for the meta-analysis. The results showed a significant difference between the 2 groups. In the experimental group, LHQW significantly improved TNF-α levels (SMD = 0.49, 95% CI: 0.16 to 0.82, *P* = .003) (Fig. 19).

#### 3.4.17. CD4^+^/CD8^+^ improvement.

**Figure 20.**
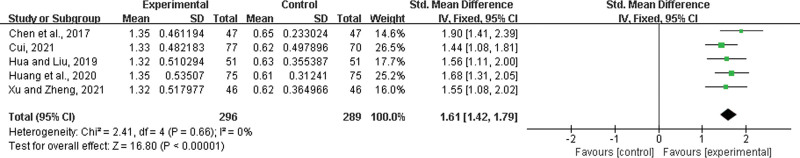
CD4+/CD8+ improvement.

Five studies^[22,23,27,28,43]^ were included, with 296 participants in the experimental group and 289 in the control group. There was no statistically significant heterogeneity among the results (*P* = .66, *I*^2^ = 0%). A fixed effects model was used for the meta-analysis. The results showed a significant difference between the 2 groups, and the experimental group had a significantly improved CD4^+^/CD8^+^ ratio (SMD = 1.61, 95% CI: 1.42 to 1.79, *P* < .00001) (Fig. 20).

### 3.5. Adverse drugs reaction

**Figure 21.**
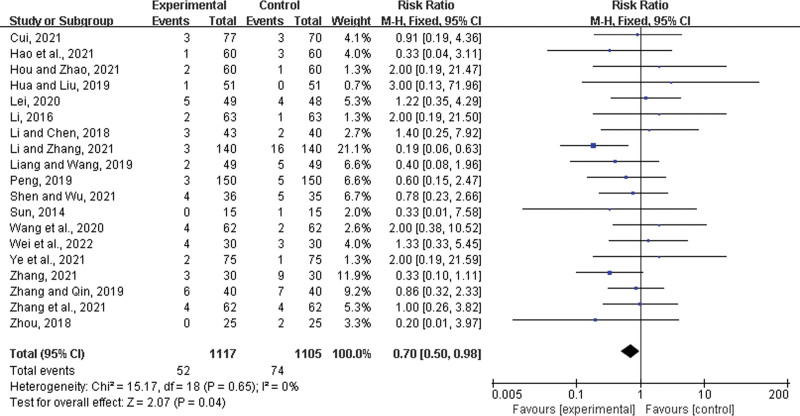
Adverse drugs reaction.

Nineteen studies^[23,25–27,30–34,37,39–42,44,48–51]^ reported ADR after therapy, mainly presenting as gastrointestinal system symptoms such as nausea and vomiting and nervous system symptoms such as dizziness; however they were relatively mild and no serious adverse events were observed. Adverse reactions in the experimental group were fewer than those in the control group (RR = 0.70, 95% CI: 0.50–0.98, *P* = .04) (Fig. 21).

### 3.6. Subgroup analysis

In our meta-analysis, subgroup analysis was performed based on age, treatment duration and form of dosage. Age was divided into 2 subgroups of children and adults. The duration of treatment was divided into 3 subgroups of 3, 5, and 7 days. The dosage of LHQW was divided into 2 subgroups of capsules and granules. As there was no heterogeneity among the subgroups, a fixed-effects model was applied (*P* > .1, *I*^2^ = 0%). The results showed that LHQW improved the clinical efficacy rate of influenza treatment in groups with different ages, different durations of treatment, and different forms of dosage (Table 2).

**Table 2:** The results of subgroup analysis.

	No. of studies	Effects model	Heterogeneity		MD	95% CI	*P* value
			*I*^2^ (%)	*P* value			
Age (total)	28	Fixed	26	.10	1.16	(1.13, 1.19)	.00001
Children	11	Fixed	11	.34	1.17	(1.12, 1.22)	.00001
Adults	17	Fixed	36	.07	1.16	(1.12, 1.20)	.00001
Duration (total)	24	Fixed	36	.04	1.17	(1.14, 1.20)	.00001
3 d	5	Random	64	.03	1.11	(1.03, 1.20)	.006
5 d	10	Fixed	0	.52	1.22	(1.16, 1.28)	.00001
7 d	9	Fixed	30	.18	1.18	(1.13, 1.23)	.00001
Dosage (total)	30	Fixed	28	.08	1.17	(1.14, 1.20)	.00001
Capsules	14	Fixed	27	.16	1.17	(1.13, 1.21)	.00001
Granules	16	Fixed	33	.10	1.17	(1.13, 1.21)	.00001

### 3.7. Publication bias

**Figure 22.**
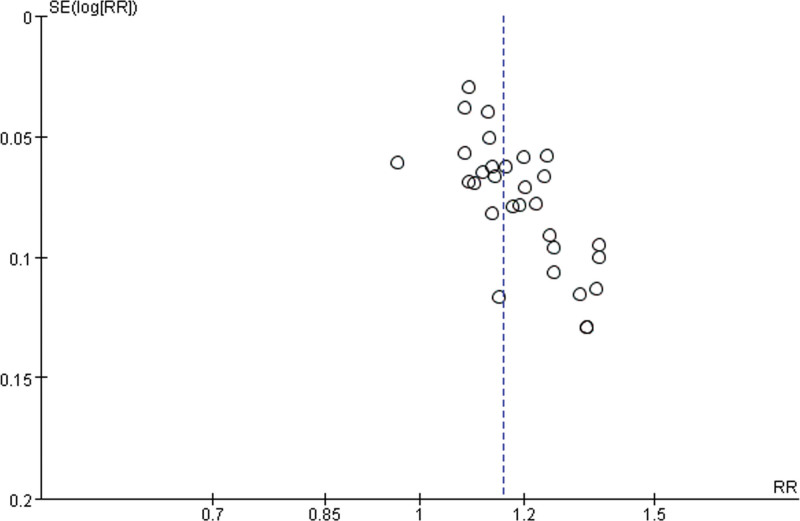
The results of publication bias.

**Figure 23.**
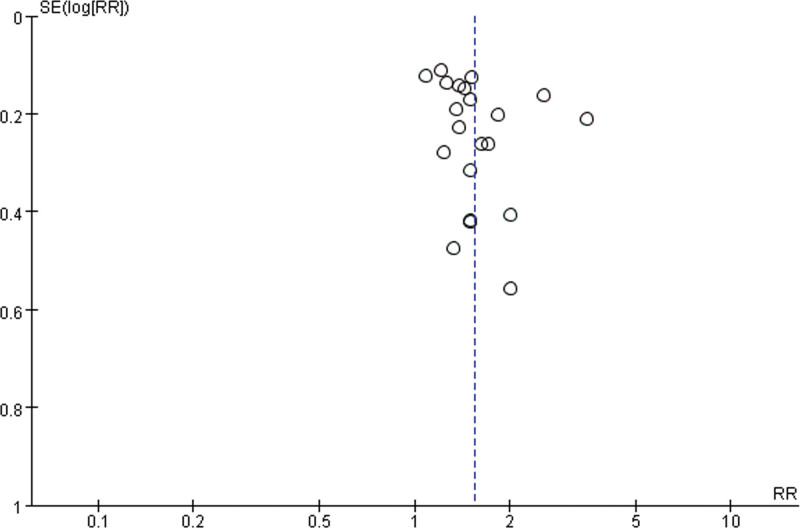
The results of publication bias.

**Figure 24:**
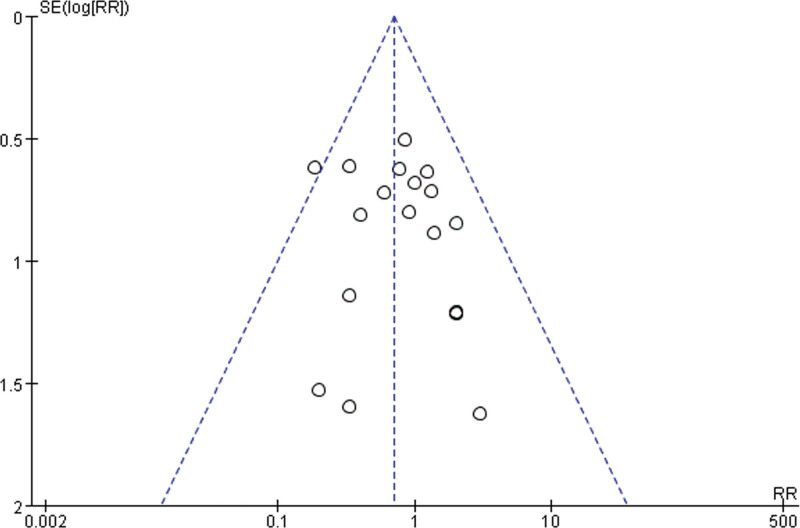
. The results of publication bias.

To evaluate the publication bias that might have been caused by this meta-analysis, funnel plots and Egger test were performed. The results showed that the funnel plots of clinical efficacy rate, cure rate, and adverse reactions were asymmetrically distributed (Figs. 22–24), suggesting that publication bias may exist. Egger test (*P* < .0001) also indicated the possible existence of publication bias.

## 4. Discussion

Influenza is an acute respiratory infectious disease caused by the influenza virus. Although the incidence of influenza symptoms varies in different regions, it is mainly characterized by systemic symptoms such as fever, headache, muscle soreness, and fatigue.^[53,54]^ Local symptoms are rare and if not treated in time may cause severe illness or even death resulting in great distress and inconvenience to patients.^[55,56]^ Influenza viruses cause cell degeneration, necrosis, and even shedding thereby spreading infection, which then leads to respiratory congestion, edema, and increased secretion, producing respiratory symptoms such as nasal congestion, runny nose, sore throat, and cough.^[57]^ Influenza viruses attack host epithelial cells to complete replication and then infect more cells, triggering the immune system to attack and destroy the infected tissues in the entire respiratory system, leading to an overreaction of the immune system.^[58]^ Influenza virus infection can lead to acute immune inflammatory damage with the involvement of inflammatory cytokines in the immune regulatory network of infection.^[59,60]^ Excessive release of pro-inflammatory cytokines such as TNF-α, IL-1, and IL-6 can lead to severe inflammatory damage in target organs^[61–63]^ and trigger a series of organ functional impairments and even multi-organ functional failure.^[64]^

LHQW has a broad-spectrum antiviral effect^[18,65]^ and can significantly inhibit influenza, severe acute respiratory syndrome,^[10]^ avian influenza,^[66,67]^ herpes simplex, and influenza A H1N1 viruses,^[12]^ which is of great value in response to epidemics. LHQW contains numerous active ingredients for the treatment of influenza,^[68]^ has the strongest killing effect on influenza and parainfluenza viruses, and effectively inhibits a variety of bacteria.^[69]^ Therefore, LHQW facilitates the treatment of bacterial and viral co-infections. With effects such as antipyretic, anti-inflammatory, relieving coughs, reducing sputum, and improving the level of cellular immunity, LHQW can effectively relieve headache, fever, sore throat, and other cold symptoms^[70]^; it can also intercept the transformation of the virus in the body and prevent the progression of cold to pneumonia and myocarditis. LHQW can regulate immunity, improve cellular and humoral immunity, and enhance the immunity of the body and its ability to recover from diseases.^[71,72]^

A large number of RCTs on LHQW adjuvant therapy for influenza were reviewed, but the sample size of a single study was small; therefore, evidence for the clinical application of LHQW are lacking. Therefore, a meta-analysis was used to systematically evaluate the existing RCTs, and the clinical efficacy rate and cure rate were considered as the main outcome indicators, and time to fever reduction and CRP improvement were considered as the secondary outcome indicators, to provide an effective basis for clinical practice. In this study, 2685 cases was analyzed and the results showed that the clinical effective rate (RR = 1.22, 95% CI: 1.18–1.26, *P* < .00001) and cure rate (RR = 1.60, 95% CI: 1.40–1.84, *P* < .00001), time to fever reduction (SMD = −2.36, 95% CI: −2.87 to −1.85, *P* < .00001), time to relief from cough (SMD = 2.06, 95% CL: −2.58 to −1.55, *P* < .00001), time to relief from sore throat (SMD = −2.16, 95% CI: −2.75 to −1.58, *P* < .00001), time to relief from muscle pain (SMD = −2.63, 95% CI: −3.79 to −1.47, *P* < .00001), relief time for runny nose (SMD = −3.72, 95% CI: −5.84 to −1.60, *P* = .0006), relief time for nasal congestion (SMD = −2.20, 95% CI: −3.50 to −0.89, *P* = .0010), headache relief time (SMD = −3.58, 95% CI: −5.81 to −1.35, *P* = .002), time to virus extinction (SMD = −0.64, 95% CI: −1.07 to −0.20, *P* = .004), time for improvement in systemic symptom (SMD = −1.51, 95% CI: −2.29 to −0.73, *P* = .0001), time for improvement in respiratory symptom (SMD = −1.09, 95% CI: −1.76 to −0.42, *P* = .001), length of hospital stay (SMD = −1.59, 95% CI: −2.30 to −0.88, *P* < .00001), CRP improvement (SMD = 1.08, 95% CI: 0.65–1.50, *P* < .00001), IL-6 improvement (SMD = 0.69, 95% CI: 0.32–1.05, *P* = .0002), TNF-α improvement (SMD = 0.49, 95% CI: 0.16–0.82, *P* = .003), and CD4^+^/CD8^+^ improvement (SMD = 1.61, 95% CI: 1.42–1.79, *P* < .00001). The LHQW for influenza was statistically significant (*P* < .01), and LHQW reduced the incidence of adverse reactions (RR = 0.70, 95% CI: 0.50–0.98, *P* = .04).

Our analysis showed that LHQW can improve the clinical effective rate and cure rate, and it has also been reported to improve the cure rate when applied to influenza virus infection,^[30,73]^ with the same therapeutic effect. In an earlier study,^[74]^ LHQW had obvious effects in clearing the plaque, detoxifying the lung, and draining heat, while our results showed that LHQW could improve fever, cough, and nasal congestion, and the results were consistent in these aspects. Another study showed that LHQW improved the release of inflammatory factors,^[14]^ which was consistent with our results that showed improved CRP, IL-6, and TNF-α levels. Therefore, LHQW adjuvant treatment for influenza may be considered clinically because of its efficacy and safety.

However, this study had several limitations. First, the literature did not describe the specific randomized method, which may produce selection bias, and details such as performance bias, detection bias, attrition bias, and reporting bias, are not adequate; further some studies on quality evaluations had high risk factors, which has an impact on the strength of evidence. Second, all the literature included in our meta-analysis were in Chinese, which may result in ethnic and regional bias. Finally, long-term prognosis (such as overall survival and recurrence rate) was not presented in this meta-analysis, making it difficult to analyze the overall efficacy and safety. Therefore, it is recommended that high-quality clinical randomized trials such as large-sample, multi-center, and double-blind RCTs should be designed and combined with the characteristics of LHQW in terms of dose administered, duration of treatment, incidence of adverse reactions, long-term treatment effects, and drug safety and efficacy to provide high-level evidence for further clinical practice.

## 5. Conclusion

The results of our meta-analysis suggest that the efficacy and safety of LHQW adjuvant therapy may be superior to those without LHQW; the clinical efficacy rate, cure rate, and many other indices are significantly improved, and the incidence of adverse reactions is reduced. Based on the results of our analysis and the theoretical basis of influenza, LHQW may be an excellent complementary and alternative therapeutic agent. However, the low quality of some studies posed a potential risk of bias, which affected the reliability of this analysis to some extent. Therefore, the long-term efficacy and safety of LHQW for influenza still needs to be confirmed by large multi-center and carefully designed rigorous RCTs to provide reliable evidence to validate the efficacy of LHQW as an adjuvant treatment for influenza.

## Acknowledgments

Thank all authors for their contributions to the paper.

## Author contributions

**Conceptualization:** Chao Yuan.

**Data curation:** Ying Guan.

**Formal analysis:** Ying Guan.

**Funding acquisition:** Ying Guan.

**Methodology:** Chao Yuan.
